# Correction: The hallmarks of childhood abuse and neglect: A systematic review

**DOI:** 10.1371/journal.pone.0296550

**Published:** 2023-12-28

**Authors:** Jason Lang, Daniel M. Kerr, Papoula Petri-Romão, Tracey McKee, Helen Smith, Naomi Wilson, Marianna Zavrou, Paul Shiels, Helen Minnis

There are errors in the Funding section. The correct Funding statement is: This study was supported by MRC- grant number MR/R004927/.
