# Supplementary material for: The hallmarks of childhood abuse and neglect: A systematic review
Source: PLoS One. 2020 Dec 8;15(12):e0243639. doi: 10.1371/journal.pone.0243639 (PMC7723263; doi:10.1371/journal.pone.0243639)
Supplement: S1 Table — (PDF) [file pone.0243639.s001.pdf]

Supplementary Table- Details of Medium Quality Articles

Citation	Year	Category	Type of abuse investigated	Review Type	Number of included studies	Number of participants in meta-analysis	Summary of result
Abajobirv[1]	2017	Social Factors	Sexual Abuse	Meta-analysis	8	38,989	Sexual abuse associated with greater risk of risky sexual behaviours in adulthood (OR=1.59, 95% CI=1.41-1.80). Overall there was no significant difference males vs females but the association was greater in females when limited to substantiated cases of abuse.
Agnew-Blais [2]	2016	Body & Mind	Combination	Meta-analysis	30	5733	Childhood maltreatment associated with greater risk of adverse outcomes in adults with bipolar affective disorder
Bayrampour [3]	2018	Body & Mind	Combination	Systematic Review	101	N/A	Childhood abuse identified as a risk factor for antenatal anxiety.
Bolen [4]	2015	Social Factors	Sexual Abuse	Meta-analysis	29	1199	Explored relationship of non-offending caregiver support and functioning after disclosure of childhood sexual abuse. No clear association identified.

Bonoldi [5]	2013	Body & Mind	Combination	Meta-analysis	23	2017	Pooled estimates of prevalence of childhood abuse in adults with psychosis was: sexual abuse=26.3%, physical abuse=38.8%, emotional abuse=34%. Significant heterogeneity identified.
Braga [6]	2018	Social Factors	Combination	Meta-Analysis	14	20,946	Childhood maltreatment associated with greater risk of adult antisocial behaviour (OR=1.96, 95% CI=1.42-2.71).
Braithwaite [7]	2017	Body & Mind	Combination	Meta-analysis	22	12,210	Childhood maltreatment associated with increased risk of depression (OR=1.50, 95% CI- 1.32-1.70). Interpersoanl relationships, cognitive difficulties and behavioural difficulties identified as possible modifiable mediators.
Bunting [8]	2018	Social Factors	Combination	Systematic Review	13	N/A	All forms of maltreatment were associated with some form of adult economic insecurity (eg.

							Unemployment, low income, sickness absence).
Butt [9]	2011	Body & Mind	Combination	Systematic Review	18	N/A	No consistent association of physical or sexual abuse with illicit drug use.
Coelho [10]	2014	Biochemical Changes	Combination	Systematic Review	20	N/A	Reviewed association of childhood maltreatment with inflammatory markers. Significant heterogeinity in study design and markers used. Strongest association for CRP
Conrad-Hiebner [11]	2018	Social Factors	Combination	Systematic Review	26	N/A	Economic insecurity (as measured by income loss, material hardship, or housing hardship) associated with greater risk of child maltreatment.
Danase [12]	2014	Body & Mind	Combination	Meta-analysis	41	190,285	Childhood maltreatment associated with greater risk of obesity throughout the life course (OR=1.36 95% CI: 1.26-1.47). Stronger association in women and white ethnicity.

Domhardt [13]	2015	Social Factors	Sexual Abuse	Systematic Review	37	N/A	10-53% of survivors of childhood sexual abuse found to be resilient (variously defined good functioning). Predictors included family and social support, education, and interpersonal and emotional competence.
Dualibe [14]	2017	Body & Mind	Combination	Systematic Review	28	N/A	Explored association of early abuse and bipolar affective disorder. Prevalence of abuse ~40% in adults with bipolar. Studies have associated abuse with early onset, rapid cycling and comorbidity.
Edalati [15]	2017	Social Factors	Combination	Systematic Review	20	N/A	Included studies identified association of childhood maltreatment with criminal justice involvement and victimisation amongst homeless adults. Association greatest for physical and sexual abuse.

Fry [16]	2012	Body & Mind	Combination	Systematic Review	106	N/A	Review of impacts of childhood abuse on children in East Asian and the Pacific. Identified increased risk of mental and physical health problems, suicide attempts, and behavioural problems.
Hauser [17]	2011	Body & Mind	Combination	Systematic Review	18	N/A	Association with physical and sexual abuse and risk of fibromyalgia identified in most studies but confounded by poor quality.
Hemmingsson [18]	2014	Body & Mind	Combination	Meta-analysis	23	112,708	Childhood abuse associated with greater risk of adult obesity (OR=1.34, 95%CI=1.24,1.45). Associated greater with increased severity of abuse.
Huang [19]	2015	Body & Mind	Combination	Meta-analysis	7	87,251	Childhood maltreatment associated with increased risk of type 2 diabetes

							(OR=1.32, 95%CI=1.16-1.51)
Hugill [20]	2017	Social Factors	Sexual Abuse	Systematic Review	14	N/A	Included studies identified an association between childhood sexual abuse and later parenting stress. Suggests that association is mostly mediating by depression, stressors, and other variables.
Hutchens [21]	2017	Body & Mind	Combination	Systematic Review	16	N/A	Majority of studies found an association of childhood abuse with greater risk of post-partum depression.
Ip [22]	2016	Body & Mind	Physical Abuse	Meta-analysis	22	17,234	Childhood physical abuse associated with increased risk of adverse mental health outcomes (OR=2.16, 95% CI=1.87-2.49). Significant heterogeneity identified.
Jespersen [23]	2009	Social Factors	Sexual Abuse	Meta-analysis	24	5095	Sexual abuse associated with increased risk of sexual offending (OR=3.36, 95%CI-2.34-4.82). Abuse also linked to

							greater risk of offences against children.
Khaleque [24]	2015	Body & Mind	Combination	Meta-analysis	33	11,755	Perceived parental neglect associated with psychological maladjustment and negative personality dispositions.
Kimber [25]	2017	Body & Mind	Emotional Abuse	Systematic Review	23	N/A	Prevalence of emotional abuse and neglect amongst adults with eating disorder ranged from 21.0% to 66.0%. Significant heterogeneity in literature.
Lang [26]	2019	Genes & Epigenomes	Combination	Systematic Review	37	N/A	There is clear evidence replicated for the important role of epigenetic modification of NR3C1 and to a lesser extend genes associated with serotonin, in association with ACE exposure. The link to telomeres is less clear. Further study of other mechanisms of epigenetic

							modification is recommended.
Li [27]	2017	Genes & Epigenomes	Combination	Meta-analysis	24	30,919	Small but significant association of childhood abuse with accelerated telomere erosion in adulthood (r=-0.05, p<0.001).
Lindert [28]	2014	Body & Mind	Combination	Meta-analysis	19	115,579	Childhood abuse associated with increased risk of depression and anxiety. Greatest effect size with sexual abuse (depression OR=2.04, 95%CI=1.65-2.53; anxiety OR=2.52 95%CI=2.12-2.98)
Liu [29]	2017	Body & Mind	Combination	Meta-analysis	16	Not stated	Childhood abuse associated with increased risk of suicidal behaviour (OR=1.36, 95%CI=1.26,1.47). This was significant for all sub-types of abuse with greatest effect size from emotional abuse

Lloyd [30]	2012	Social Factors	Sexual Abuse	Meta-analysis	12	15,622	Amongst men who have sex with men, childhood sexual abuse associated with greater risk of risky sexual behaviours and higher rates of HIV infection
Mandelli [31]	2015	Body & Mind	Combination	Meta-analysis	26	35,772	Childhood maltreatment associated with risk of depression in adulthood. Highest effect size with neglect.
Maniglio [32]	2015	Social Factors	Sexual Abuse	Systematic Review	36	N/A	Childhood sexual abuse associated with conduct disorder in all studies and remained significant after adjusting for confounders. ORs ranged from 2-12.
Maniglio [33]	2013	Body & Mind	Sexual Abuse	Systematic Review	4	N/A	Childhood sexual abuse associated with increased risk for all anxiety disorders, particularly PTSD. No difference by gender or abuse severity.
Maniglio [34]	2013	Body & Mind	Sexual Abuse	Systematic Review	18	N/A	Explored association of childhood sexual abuse with outcomes in bipolar

							affective disorder. CSA associated with with comorbid PTSD, inconsistent findings relating to severity markers and comorbidity.
McTavish [35]	2019	Body & Mind	Sexual Abuse	Systematic Review	22	N/A	Highlights limitations in literature around disclosure in PTSD with lack of longitudinal data a particular concern.
Midei [36]	2011	Body & Mind	Combination	Systematic Review	36	138,746	Childhood sexual abuse and physical abuse associated with obesity. Suggests pathway mediated by disordered eating and emotional dysregulation rather than reduced physical activity.
Molendijk [37]	2017	Body & Mind	Combination	Meta-analysis	82	35,887	Higher rates of childhood maltreatment in adults with eating disorders compared to other mental illnesses (OR=1.31, 95%CI=1.08-1.58). Childhood maltreatment associated with eating

							disorder severity and comorbidity.
Montgomery [38]	2019	Social Factors	Combination	Systematic Review	15	N/A	Prevalence of child abuse increased in populations exposed to traumatic events. Range of trauma exposures included childhood abuse, genocide, warfare etc
Nanni [39]	2012	Body & Mind	Combination	Meta-analysis	26	26,642	Childhood maltreatment associated with risk of recurrent and persistent depressive episodes (OR=2.27, 95%CI=1.80-2.87) and with risk of treatment resistance (OR=1.43, 95%CI-1.11-1.83).
Naughton [40]	2013	Body & Mind	Combination	Systematic Review	42	N/A	Review of emotional, behavioural and developmental features of neglect and/or emotional abuse in pre-school children. Identified variety of features including attachment style, cognitive

							development, and peer relations.
Rafiq [41]	2018	Body & Mind	Combination	Meta-analysis	30	2199	Moderate association of childhood abuse and dissociative experience in adults with severe mental illnesses (r=0.33, p<0.001).
Rhodes [42]	2011	Body & Mind	Sexual Abuse	Systematic Review	16	N/A	Childhood Sexual Abuse associated with risk of suicide, effect may be greater in boys.
Schneeberger [43]	2014	Social Factors	Combination	Systematic Review	73	N/A	LGBT populations report greater rates of childhood maltreatment of all types.
Sonneveld [44]	2013	Body & Mind	Sexual Abuse	Systematic Review	2	N/A	Prevalence of sexual abuse in children with chronic abdominal pain ranged from 2-8%.
Varese [45]	2012	Body & Mind	Combination	Meta-analysis	41	81,253	Childhood abuse and neglect associated with increased risk of psychosisOR=2.89, 95%CI=2.34-3.31)
Viola [46]	2016	Social Factors	Combination	Meta-analysis	288	59,692	CTQ scores were significantly lower in

							Europe and Asia compared to other continents (particularly South America). Association identified of greater GDP with lower mean CTQ scores in population.

1- Abajobir, A. A., Kisely, S., Maravilla, J. C., Williams, G., & Najman, J. M. (2017). Gender differences in the association between childhood sexual abuse and risky sexual behaviours: A systematic review and meta-analysis. *Child Abuse & Neglect, 63*, 249-260. doi:https://dx.doi.org/10.1016/j.chiabu.2016.11.023

2- AgnewBlais, J., & Danese, A. (2016). Childhood maltreatment and unfavourable clinical outcomes in bipolar disorder: A systematic review and meta-analysis. *The Lancet Psychiatry, 3*(4), 342-349. doi:http://dx.doi.org/10.1016/S2215-0366%2815%2900544-1

3- Bayrampour, H., Vinturache, A., Hetherington, E., Lorenzetti, D. L., & Tough, S. (2018). Risk factors for antenatal anxiety: A systematic review of the literature. *Journal of Reproductive & Infant Psychology, 36*(5), 476-503. doi:https://dx.doi.org/10.1080/02646838.2018.1492097

4- Bolen, R. M., & Gergely, K. B. (2015). A meta-analytic review of the relationship between non-offending caregiver support and post-disclosure functioning in sexually abused children. *Trauma, Violence, & Abuse, 16*(3), 258-279. doi:http://dx.doi.org/10.1177/1524838014526307

5- Bonoldi, I., Simeone, E., Rocchetti, M., Codjoe, L., Rossi, G., Gambi, F., Fusar-Poli, P. (2013). Prevalence of self-reported childhood abuse in psychosis: A meta-analysis of retrospective studies. *Psychiatry Research, 210*(1), 8-15. doi:http://dx.doi.org/10.1016/j.psychres.2013.05.003

6- Braga T., Cunha O., & Maia, A. (2018). The enduring effect of maltreatment on antisocial behavior: A meta-analysis of longitudinal studies. *Aggression and Violent Behavior, 40*, 91-100. doi:http://dx.doi.org/10.1016/j.avb.2018.04.003

7- Braithwaite, E. C., O'Connor, R. M., Degli-Esposti, M., Luke, N., & Bowes, L. (2017). Modifiable predictors of depression following childhood maltreatment: A systematic review and meta-analysis. *Transl Psychiatry Psychiatry, 7*(7), e1162. doi:https://dx.doi.org/10.1038/tp.2017.140

8- Bunting, L., Davidson, G., McCartan, C., Hanratty, J., Bywaters, P., Mason, W., & Steils, N. (2018). The association between child maltreatment and adult poverty-A systematic review of longitudinal research. *Child Abuse & Neglect*, *77*, 121-133. doi:http://dx.doi.org/10.1016/j.chiabu.2017.12.022

9- Butt, S., Chou, S., & Browne, K. (2011). A rapid systematic review on the association between childhood physical and sexual abuse and illicit drug use among males. *Child Abuse Review*, *20*(1), 6-38. doi:http://dx.doi.org/10.1002/car.1100

10- Coelho, R., Viola, T. W., WalssBass, C., Brietzke, E., & GrassiOliveira, R. (2014). Childhood maltreatment and inflammatory markers: A systematic review. *Acta Psychiatrica Scandinavica*, *129*(3), 180-192. doi:http://dx.doi.org/10.1111/acps.12217

11- Conrad-Hiebner, A., & Byram, E. (2018). The temporal impact of economic insecurity on child maltreatment: A systematic review. *Trauma Violence & Abuse, 1524838018756122* doi:https://dx.doi.org/10.1177/1524838018756122

12- Danese, A., & Tan, M. (2014). Childhood maltreatment and obesity: Systematic review and meta-analysis. *Molecular Psychiatry*, *19*(5), 544-554. doi:https://dx.doi.org/10.1038/mp.2013.54

13- Domhardt, M., Munzer, A., Fegert, J. M., & Goldbeck, L. (2015). Resilience in survivors of child sexual abuse: A systematic review of the literature. *Trauma, Violence, & Abuse, 16*(4), 476-493. doi:http://dx.doi.org/10.1177/1524838014557288

14- Dualibe, A. L., & Osorio, F. L. (2017). Bipolar disorder and early emotional trauma: A critical literature review on indicators of prevalence rates and clinical outcomes. *Harvard Review of Psychiatry, 25*(5), 198-208. doi:http://dx.doi.org/10.1097/HRP.0000000000000154

15- Edalati, H., & Nicholls, T. L. (2017). Childhood maltreatment and the risk for criminal justice involvement and victimization among homeless individuals: A systematic review. *Trauma Violence & Abuse, 1524838017708783* doi:https://dx.doi.org/10.1177/1524838017708783

16- Fry, D., McCoy, A., & Swales, D. (2012). The consequences of maltreatment on children's lives: A systematic review of data from the east asia and pacific region. *Trauma Violence & Abuse, 13*(4), 209-233. doi:https://dx.doi.org/10.1177/1524838012455873

17- Hauser, W., Kosseva, M., Uceyler, N., Klose, P., & Sommer, C. (2011). Emotional, physical, and sexual abuse in fibromyalgia syndrome: A systematic review with meta-analysis. *Arthritis Care & Research, 63*(6), 808-820. doi:https://dx.doi.org/10.1002/acr.20328

18- Hemmingsson, E., Johansson, K., & Reynisdottir, S. (2014). Effects of childhood abuse on adult obesity: A systematic review and meta-analysis. *Obesity Reviews*, *15*(11), 882-893. doi:https://dx.doi.org/10.1111/obr.12216

19- Huang, H., Yan, P., Shan, Z., Chen, S., Li, M., Luo, C., Liu, L. (2015). Adverse childhood experiences and risk of type 2 diabetes: A systematic review and meta-analysis. *Metabolism: Clinical & Experimental, 64*(11), 1408-1418. doi:https://dx.doi.org/10.1016/j.metabol.2015.08.019

20- Hugill, M., Berry, K., & Fletcher, I. (2017). The association between historical childhood sexual abuse and later parenting stress: A systematic review. *Archives of Women's Mental Health, 20*(2), 257-271. doi:http://dx.doi.org/10.1007/s00737-016-0708-3

21- Hutchens, B. F., Kearney, J., & Kennedy, H. P. (2017). Survivors of child maltreatment and postpartum depression: An integrative review. *Journal of Midwifery & Women's Health, 62*(6), 706-722. doi:http://dx.doi.org/10.1111/jmwh.12680

22- Ip, P., Wong, R. S., Li, S. L., Chan, K. L., Ho, F. K., & Chow, C. (2016). Mental health consequences of childhood physical abuse in chinese populations: A meta-analysis. *Trauma, Violence, & Abuse, 17*(5), 571-584. doi:http://dx.doi.org/10.1177/1524838015585317

23- Jespersen, A. F., Lalumiere, M. L., & Seto, M. C. (2009). Sexual abuse history among adult sex offenders and non-sex offenders: A meta-analysis. *Child Abuse & Neglect, 33*(3), 179-192. doi:http://dx.doi.org/10.1016/j.chiabu.2008.07.004

24- Khaleque, A. (2015). Perceived parental neglect, and children's psychological maladjustment, and negative personality dispositions: A meta-analysis of multi-cultural studies. *Journal of Child and Family Studies, 24*(5), 1419-1428. doi:http://dx.doi.org/10.1007/s10826-014-9948-x

25- Kimber, M., McTavish, J. R., Couturier, J., Boven, A., Gill, S., Dimitropoulos, G., & MacMillan, H. L. (2017). Consequences of child emotional abuse, emotional neglect and exposure to intimate partner violence for eating disorders: A systematic critical review. *BMC Psychology, 5,* Art 33-18. doi:http://dx.doi.org/10.1186/s40359-017-0202-3

26- Lang, J., McKie, J., Smith, H., McLaughlin, A., Gillberg, C., Shiels, P. G., & Minnis, H. (2019). Adverse childhood experiences, epigenetics and telomere length variation in

childhood and beyond: A systematic review of the literature. *European Child & Adolescent Psychiatry*, doi:https://dx.doi.org/10.1007/s00787-019-01329-1

27- Li, X., Wang, J., Zhou, J., Huang, P., & Li, J. (2017). The association between post-traumatic stress disorder and shorter telomere length: A systematic review and meta-analysis. *Journal of Affective Disorders*, *218*, 322-326. doi:http://dx.doi.org/10.1016/j.jad.2017.03.048

28- Lindert, J., von Ehrenstein, O. S., Grashow, R., Gal, G., Braehler, E., & Weisskopf, M. G. (2014). Sexual and physical abuse in childhood is associated with depression and anxiety over the life course: Systematic review and meta-analysis. *International Journal of Public Health*, *59*(2), 359-372. doi:http://dx.doi.org/10.1007/s00038-013-0519-5

29- Liu, J., Fang, Y., Gong, J., Cui, X., Meng, T., Xiao, B., Luo, X. (2017). Associations between suicidal behavior and childhood abuse and neglect: A meta-analysis. *Journal of Affective Disorders, 220*, 147-155. doi:http://dx.doi.org/10.1016/j.jad.2017.03.060

30- Lloyd, S., & Operario, D. (2012). HIV risk among men who have sex with men who have experienced childhood sexual abuse: Systematic review and meta-analysis. *AIDS Education and Prevention, 24*(3), 228-241. doi:http://dx.doi.org/10.1521/aeap.2012.24.3.228

31- Mandelli, L., Petrelli, C., & Serretti, A. (2015). The role of specific early trauma in adult depression: A meta-analysis of published literature. childhood trauma and adult depression. *European Psychiatry, 30*(6), 665-680. doi:http://dx.doi.org/10.1016/j.eurpsy.2015.04.007

32- Maniglio, R. (2013). Child sexual abuse in the etiology of anxiety disorders: A systematic review of reviews. *Trauma Violence & Abuse, 14*(2), 96-112. doi:https://dx.doi.org/10.1177/1524838012470032

33- Maniglio, R. (2013). The impact of child sexual abuse on the course of bipolar disorder: A systematic review. *Bipolar Disorders, 15*(4), 341-358. doi:http://dx.doi.org/10.1111/bdi.12050

34- Maniglio, R. (2015). Significance, nature, and direction of the association between child sexual abuse and conduct disorder: A systematic review. *Trauma Violence & Abuse, 16*(3), 241-257. doi:https://dx.doi.org/10.1177/1524838014526068

35- McTavish, J. R., Sverdlichenko, I., MacMillan, H. L., & Wekerle, C. (2019). Child sexual abuse, disclosure and PTSD: A systematic and critical review. *Child Abuse & Neglect, 92*, 196-208. doi:https://dx.doi.org/10.1016/j.chiabu.2019.04.006

36- Midei, A. J., & Matthews, K. A. (2011). Interpersonal violence in childhood as a risk factor for obesity: A systematic review of the literature and proposed pathways. *Obesity Reviews, 12*(5), e159-72. doi:https://dx.doi.org/10.1111/j.1467-789X.2010.00823.x

37- Molendijk, M. L., Hoek, H. W., Brewerton, T. D., & Elzinga, B. M. (2017). Childhood maltreatment and eating disorder pathology: A systematic review and dose-response meta-analysis. *Psychological Medicine, 47*(8), 1402-1416. doi:http://dx.doi.org/10.1017/S0033291716003561

38- Montgomery, E., Just-Ostergaard, E., & Jervelund, S. S. (2019). Transmitting trauma: A systematic review of the risk of child abuse perpetrated by parents exposed to traumatic events. *International Journal of Public Health, 64*(2), 241-251. doi:https://dx.doi.org/10.1007/s00038-018-1185-4

39- Nanni, V., Uher, R., & Danese, A. (2012). Childhood maltreatment predicts unfavorable course of illness and treatment outcome in depression: A meta-analysis. *The American Journal of Psychiatry*, *169*(2), 141-151. doi:http://dx.doi.org/10.1176/appi.ajp.2011.11020335

40- Naughton, A. M., Maguire, S. A., Mann, M. K., Lumb, R. C., Tempest, V., Gracias, S., & Kemp, A. M. (2013). Emotional, behavioral, and developmental features indicative of neglect or emotional abuse in preschool children: A systematic review. *JAMA Pediatrics, 167*(8), 769-775. doi:https://dx.doi.org/10.1001/jamapediatrics.2013.192

41- Rafiq S., Campodonico C., & Varese, F. (2018). The relationship between childhood adversities and dissociation in severe mental illness: A meta-analytic review. *Acta Psychiatrica Scandinavica, 138*(6), 509-525. doi:http://dx.doi.org/10.1111/acps.12969

42- Rhodes, A. E., Boyle, M. H., Tonmyr, L., Wekerle, C., Goodman, D., Leslie, B., Manion, I. (2011). Sex differences in childhood sexual abuse and suicide-related behaviors. *Suicide & Life-Threatening Behavior, 41*(3), 235-254. doi:https://dx.doi.org/10.1111/j.1943-278X.2011.00025.x

43- Schneeberger, A. R., Dietl, M. F., Muenzenmaier, K. H., Huber, C. G., & Lang, U. E. (2014). Stressful childhood experiences and health outcomes in sexual minority populations: A systematic review. *Social Psychiatry & Psychiatric Epidemiology*, *49*(9), 1427-1445. doi:https://dx.doi.org/10.1007/s00127-014-0854-8

44- Sonneveld L.P., BrilleslijperKater S.N., Benninga M.A., Van Konijnenburg E.M.M.H., SieswerdaHoogendoorn T., & Teeuw, A. H. (2013). Prevalence of child sexual abuse in pediatric patients with chronic abdominal pain. *Journal of Pediatric Gastroenterology and Nutrition, 56*(5), 475-480. doi:http://dx.doi.org/10.1097/MPG.0b013e31828b5145

45- Varese, F., Smeets, F., Drukker, M., Lieverse, R., Lataster, T., Viechtbauer, W., . . . Bentall, R. P. (2012). Childhood adversities increase the risk of psychosis: A meta-analysis of patient-control, prospective- and cross-sectional cohort studies. *Schizophrenia Bulletin, 38*(4), 661-671. doi:http://dx.doi.org/10.1093/schbul/sbs050

46- Viola, T. W., Salum, G. A., KluweSchiavon, B., SanvicenteVieira, B., Levandowski, M. L., & GrassiOliveira, R. (2016). The influence of geographical and economic factors in estimates of childhood abuse and neglect using the childhood trauma questionnaire: A worldwide meta-regression analysis. *Child Abuse & Neglect, 51*, 1-11. doi:http://dx.doi.org/10.1016/j.chiabu.2015.11.019
