# Supplementary material for: The hallmarks of childhood abuse and neglect: A systematic review
Source: PLoS One. 2020 Dec 8;15(12):e0243639. doi: 10.1371/journal.pone.0243639 (PMC7723263; doi:10.1371/journal.pone.0243639)
Supplement: S2 File — (PDF) [file pone.0243639.s003.pdf]

Detailed Search Terms:

Database: Ovid MEDLINE(R) ALL <1946 to May 28, 2019>

Search Strategy:

----

1 Child Abuse/ (21075)

2 Child Abuse, Sexual/ (9724)

3 "Adult Survivors of Child Abuse"/ (1958)

4 Adverse Childhood Experiences/ (163)

5 ((child* or early) adj3 (abuse* or neglect* or molest* or maltreat* or advers* or stress* or trauma*)).tw. (51206)

6 socioeconomic factors/ or economic status/ or poverty/ or poverty areas/ or social class/ or social mobility/ or cultural deprivation/ or public assistance/ or social security/ or aid to families with dependent children/ (217348)

7 educational status/ or academic failure/ or literacy/ or employment/ or unemployment/ or family characteristics/ or marital status/ or divorce/ or marriage/ or single parent/ or widowhood/ or income/ (161031)

8 gender identity/ or femininity/ or masculinity/ or men/ or women/ (33573)

9 child, abandoned/ or child, adopted/ or "child of impaired parents"/ or child, foster/ or child, orphaned/ or child, unwanted/ or Disabled Children/ (12323)

10 "sexual and gender minorities"/ or transgender persons/ or sexuality/ or bisexuality/ or homosexuality/ or homosexuality, female/ or homosexuality, male/ or transsexualism/ (39331)

11 education/ or educational measurement/ or academic performance/ or academic success/ (56452)

12 loneliness/ or love/ or Social Isolation/ (18597)

13 exp Ethnic Groups/ or Minority Groups/ (152407)

14 "Transients and Migrants"/ or human migration/ or "emigration and immigration"/ (33059)

15 social environment/ or community networks/ or social support/ or psychosocial support systems/ (110511)

16 residence characteristics/ or housing/ or public housing/ or social capital/ or Built Environment/ (48692)

17 ((socioeconomic or socio economic) adj3 (factor* or status)).tw. (54634)

18 (poverty or poor or depriv* or employ* or income*).tw. (1222744)

19 (gender or men or women or man or woman or girl* or boy* or disabilit* or disabled or LGBT* or lesbian* or gay or bisexual* or transgender or homosexual* or transwomen or transwoman or transmen or transman or transpeople or transperson*).tw. (2135667)

20 (educat* or school* or marital status or marriage or married or divorc* or estranged or lonel* or isolat*).tw. (2037903)

21 ((romantic or intimate) adj3 (partner* or relationship*)).tw. (13705)

22 (ethnic* or minorit* or migrant* or migration or immigrant* or immigration).tw. (434435)

23 (neighbourhood* or living conditions or housing).tw. (39129)

24 (social adj3 (capital or support or condition*)).tw. (46605)

25 exp Genetics/ or exp Genetic Phenomena/ or Epigenomics/ or DNA Methylation/ or exp Biomarkers/ (4000199)

26 (gene or genetic* or genotype* or polymorphism or gene-environment interaction* or epigen* or DNA methylation or biochemical marker* or biomarker*).tw. (2535546)

27 exp Mental Disorders/ or exp Substance-Related Disorders/ or self-injurious behavior/ or self mutilation/ or suicide/ or suicidal ideation/ or suicide, attempted/ or exp Sleep Wake Disorders/ (1262823)

28 (depressi* or panic or suicid* or self harm* or ((substance* or drug* or alcohol) adj3 (misus* or abus* or disorder*)) or sleep disturbance* or sleep disorder* or PTSD or CPTSD or mood disorder* or anxiety or agoraphobia or GAD or oppositional defiant disorder or conduct disorder* or eating disorder* or bipolar).tw. (683691)

29 exp Obesity/ or exp Smoking/ or exp Pulmonary Disease, Chronic Obstructive/ or exp Heart Diseases/ or exp Gastrointestinal Diseases/ or exp Diabetes Mellitus/ or exp Kidney Diseases/ (3076970)

30 (obese or obesity or smoking or smoker* or COPD or heart disease* or gastro-intestinal disorder* or diabet* or renal disease*).tw. (1176498)

31 exp Autonomic Nervous System/ or exp Arrhythmia, Sinus/ or Heart Rate/ or Galvanic Skin Response/ (288073)

32 (((stress or cardiovascular) adj (respons* or react*)) or autonomic nervous system or heart rate varia* or sinus arrhythmia or pre-ejection period or heart rate or vagal or sympathetic or parasympathetic or skin conductance or galvanic skin response or GSR or SCL or SCR).tw. (313430)

33 exp Neuroimaging/ or exp Magnetic Resonance Imaging/ (524046)

34 ((Brain adj3 imag*) or magnetic resonance imaging or MRI or fMRI or neuroimaging).tw. (391863)

35 exp Hydrocortisone/ or exp Inflammation/ or exp Immune System/ or exp Cytokines/ or C-Reactive Protein/ or exp Acute-Phase Proteins/ or exp NF-kappa B/ or exp Interleukins/ or

exp Fibrinogen/ or exp Leukocytes/ or exp Lymphocytes/ or Tumor Necrosis Factor-alpha/ (1955356)

36 (cortisol or HPA axis or hypothalamic pituitary adrenal axis or Inflammation or Immune System or Cytokine* or C-Reactive Protein or Acute Phase Proteins or NF-kappa B or Interleukins or Fibrinogen or White Blood Cell* or White Cell* or Leukocyte* or Lymphocyte* or TNF-alpha).tw. (1276847)

37 1 or 2 or 3 or 4 or 5 (63482)

38 6 or 7 or 8 or 9 or 10 or 11 or 12 or 13 or 14 or 15 or 16 or 17 or 18 or 19 or 20 or 21 or 22 or 23 or 24 (5501977)

39 25 or 26 (4809139)

40 27 or 28 (1624523)

41 29 or 30 (3488788)

42 31 or 32 (469343)

43 33 or 34 (670417)

44 35 or 36 (2459967)

45 38 or 39 or 40 or 41 or 42 or 43 or 44 (14271959)

46 37 and 45 (44465)

47 limit 46 to english language (41662)

48 (MEDLINE or systematic review).tw. or meta analysis.pt. (241425)

49 47 and 48 (897)

50 limit 49 to yr="2009 -Current" (732)

****
