# Supplementary material for: The hallmarks of childhood abuse and neglect: A systematic review
Source: PLoS One. 2020 Dec 8;15(12):e0243639. doi: 10.1371/journal.pone.0243639 (PMC7723263; doi:10.1371/journal.pone.0243639)
Supplement: Attachment — Submitted filename: PONE-D-20-08783_reviewer_05_22_2020_LM.pdf [file pone.0243639.s005.pdf]

PLOS ONE

The hallmarks of childhood abuse and neglect: a systematic review

--Manuscript Draft--

**Manuscript Number:**	PONE-D-20-08783
**Article Type:**	Research Article
**Full Title:**	The hallmarks of childhood abuse and neglect: a systematic review
**Short Title:**	The hallmarks of childhood abuse and neglect
**Corresponding Author:**	Daniel Kerr, MBChB, MRCPsych<br>University of Glasgow School of Medicine Dentistry and Nursing<br>Glasgow, UNITED KINGDOM
**Keywords:**	Child Abuse; Child Neglect; Risk Factors; Outcomes; Hallmarks
**Abstract:**	Background : Studies on the impacts of childhood abuse and neglect have been conducted in diverse areas. Mechanistic understanding of the complex interplay between factors is lacking. Hallmarking is an approach which identifies common factors across studies and highlights the most robust findings.<br><br>Objectives: In a review of systematic reviews and meta-analyses, we addressed the following questions: 1) What are the hallmarks of exposure to childhood abuse and neglect across the bio-ecological spectrum? 2) What is the strength of evidence to support each hallmark? 3) What are the gaps that future research should address?<br><br>Methods: A comprehensive literature search was carried out to find relevant systematic reviews or meta-analyses. 269 articles were read in full and 178 articles, encompassing more than 6000 original papers, were included in the final synthesis. All reviews were independently rated for quality by at least 2 reviewers.<br><br>Results: Of 178 review articles, 6 were rated as high quality (all meta-analyses) and 46 were rated as medium quality. Based on the most commonly reported high quality findings we propose that the hallmarks of child abuse and neglect are: Increased risk of psychopathology; Increased risk of obesity; Increased risk of high- risk sexual behaviours , Increased risk of smoking, and Increased risk of abuse and neglect in children with disabilities.<br><br>Conclusions: Hallmarks of child abuse and neglect were identified. Research gaps include a lack of focus on complexity and resilience. Adequately powered prospective studies are required to move the field forward.
**Order of Authors:**	Jason Lang
	Daniel Michael Kerr, MBChB, MRCPsych
	Papoula Petri
	Tracey McKee
	Helen Smith
	Naomi Wilson
	Marianna Zavrou
	Paul Shiels
	Helen Minnis
**Additional Information:**	
**Question**	**Response**
**Financial Disclosure**<br><br>Enter a financial disclosure statement that	The authors received no specific funding for this work.

Powered by Editorial Manager® and ProduXion Manager® from Aries Systems Corporation

describes the sources of funding for the work included in this submission. Review the submission guidelines for detailed requirements. View published research articles from *PLOS ONE* for specific examples.<br><br>This statement is required for submission and **will appear in the published article** if the submission is accepted. Please make sure it is accurate.<br><br>**Unfunded studies**<br>Enter: *The author(s) received no specific funding for this work.*<br><br>**Funded studies**<br>Enter a statement with the following details:<br>• Initials of the authors who received each award<br>• Grant numbers awarded to each author<br>• The full name of each funder<br>• URL of each funder website<br>• Did the sponsors or funders play any role in the study design, data collection and analysis, decision to publish, or preparation of the manuscript?<br>• **NO** - Include this sentence at the end of your statement: *The funders had no role in study design, data collection and analysis, decision to publish, or preparation of the manuscript.*<br>• **YES** - Specify the role(s) played.<br><br>* typeset	
**Competing Interests**<br><br>Use the instructions below to enter a competing interest statement for this submission. On behalf of all authors, disclose any competing interests that could be perceived to bias this work—acknowledging all financial support and any other relevant financial or non-financial competing interests.<br><br>This statement **will appear in the published article** if the submission is accepted. Please make sure it is accurate. View published research articles from *PLOS ONE* for specific examples.	The authors have declared no competing interests exist.

Powered by Editorial Manager® and ProduXion Manager® from Aries Systems Corporation

**NO authors have competing interests**<br><br>Enter: *The authors have declared that no competing interests exist.*<br><br>**Authors with competing interests**<br><br>Enter competing interest details beginning with this statement:<br><br>*I have read the journal's policy and the authors of this manuscript have the following competing interests: [insert competing interests here]*<br><br>* typeset	
**Ethics Statement**<br><br>Enter an ethics statement for this submission. This statement is required if the study involved:<br><br>• Human participants<br>• Human specimens or tissue<br>• Vertebrate animals or cephalopods<br>• Vertebrate embryos or tissues<br>• Field research<br><br>Write "N/A" if the submission does not require an ethics statement.<br><br>General guidance is provided below. Consult the submission guidelines for detailed instructions. **Make sure that all information entered here is included in the Methods section of the manuscript.**	N/A

Powered by Editorial Manager® and ProduXion Manager® from Aries Systems Corporation

**Format for specific study types**<br><br>**Human Subject Research (involving human participants and/or tissue)**<br>• Give the name of the institutional review board or ethics committee that approved the study<br>• Include the approval number and/or a statement indicating approval of this research<br>• Indicate the form of consent obtained (written/oral) or the reason that consent was not obtained (e.g. the data were analyzed anonymously)<br><br>**Animal Research (involving vertebrate animals, embryos or tissues)**<br>• Provide the name of the Institutional Animal Care and Use Committee (IACUC) or other relevant ethics board that reviewed the study protocol, and indicate whether they approved this research or granted a formal waiver of ethical approval<br>• Include an approval number if one was obtained<br>• If the study involved *non-human primates*, add *additional details* about animal welfare and steps taken to ameliorate suffering<br>• If anesthesia, euthanasia, or any kind of animal sacrifice is part of the study, include briefly which substances and/or methods were applied<br><br>**Field Research**<br><br>Include the following details if this study involves the collection of plant, animal, or other materials from a natural setting:<br>• Field permit number<br>• Name of the institution or relevant body that granted permission	
**Data Availability**<br><br>Authors are required to make all data underlying the findings described fully available, without restriction, and from the time of publication. PLOS allows rare exceptions to address legal and ethical concerns. See the PLOS Data Policy and FAQ for detailed information.	Yes - all data are fully available without restriction

Powered by Editorial Manager® and ProduXion Manager® from Aries Systems Corporation

A Data Availability Statement describing where the data can be found is required at submission. Your answers to this question constitute the Data Availability Statement and **will be published in the article**, if accepted.<br><br>**Important:** Stating 'data available on request from the author' is not sufficient. If your data are only available upon request, select 'No' for the first question and explain your exceptional situation in the text box.<br><br>Do the authors confirm that all data underlying the findings described in their manuscript are fully available without restriction?	
**Describe where the data may be found in full sentences. If you are copying our sample text, replace any instances of XXX with the appropriate details.**<br><br>• If the data are **held or will be held in a public repository**, include URLs, accession numbers or DOIs. If this information will only be available after acceptance, indicate this by ticking the box below. For example: *All XXX files are available from the XXX database (accession number(s) XXX, XXX.*).<br>• If the data are all contained **within the manuscript and/or Supporting Information files**, enter the following: *All relevant data are within the manuscript and its Supporting Information files.*<br>• If neither of these applies but you are able to provide **details of access elsewhere**, with or without limitations, please do so. For example:<br><br>*Data cannot be shared publicly because of [XXX]. Data are available from the XXX Institutional Data Access / Ethics Committee (contact via XXX) for researchers who meet the criteria for access to confidential data.*<br><br>*The data underlying the results presented in the study are available from (include the name of the third party*	All relevant data are within the manuscript and its Supporting Information files.

and contact information or URL).<br>• This text is appropriate if the data are owned by a third party and authors do not have permission to share the data.<br><br>* typeset	
Additional data availability information:	

Click here to download Manuscript Hallmarks of Childhood Abuse and Neglect PLOS Draft.docx

The hallmarks of childhood abuse and neglect: a systematic review

Jason Lang[1,2], Daniel M Kerr[1*], Papoula Petri[1], Tracey McKee[3], Helen Smith[3], Naomi Wilson[3], Marianna Zavrou[3], Paul Shiels[1¶], Helen Minnis[1¶]

1- University of Glasgow. Glasgow. United Kingdom.
2- NHS Lanarkshire. Lanarkshire. United Kingdom
3- NHS Greater Glasgow and Clyde. Glasgow. United Kingdom.

¶- These authors contributed equally to the work

*Corresponding Author

Daniel.Kerr@glasgow.ac.uk (DMK)

Declarations of Interests: None
Funding: This research did not receive any specific grant from funding agencies in the public, commercial, or not-for-profit sectors

Abstract

Background: Studies on the impacts of childhood abuse and neglect have been conducted in diverse areas. Mechanistic understanding of the complex interplay between factors is lacking. Hallmarking is an approach which identifies common factors across studies and highlights the most robust findings.

Objectives: In a review of systematic reviews and meta-analyses, we addressed the following questions: 1) What are the hallmarks of exposure to childhood abuse and neglect across the bio-ecological spectrum? 2) What is the strength of evidence to support each hallmark? 3) What are the gaps that future research should address?

Methods: A comprehensive literature search was carried out to find relevant systematic reviews or meta-analyses. 269 articles were read in full and 178 articles, encompassing more than 6000 original papers, were included in the final synthesis. All reviews were independently rated for quality by at least 2 reviewers.

Results: Of 178 review articles, 6 were rated as high quality (all meta-analyses) and 46 were rated as medium quality. Based on the most commonly reported high quality findings we propose that the hallmarks of child abuse and neglect are: *Increased risk of psychopathology; Increased risk of obesity; Increased risk of high- risk sexual behaviours*, *Increased risk of smoking,* and *Increased risk of abuse and neglect in children with disabilities.*

Conclusions: Hallmarks of child abuse and neglect were identified. Research gaps include a lack of focus on complexity and resilience. Adequately powered prospective studies are required to move the field forward.

Introduction

In the two decades since the seminal publication of the CDC/Kaiser Permanente Adverse Childhood Experiences (ACE) Study [1], there has been a growing interest in the long term effects of exposure to childhood abuse and neglect and the long-term impact such exposure may confer on individuals so exposed. ACE exposure and its association with impaired biological and psychosocial functioning, has found traction in both the popular press and with governments around the world [2, 3].

The history of the development of this literature has been summarised by several authors in the field [4, 5]. Certain recurrent findings have been noted in the literature in relation to associations between exposure to child abuse and neglect and the development of physical, psychological, and social problems in adulthood [4, 5]. This literature has become increasingly broad as additional concepts have been discovered and documented. An example of this is in the area of epigenetics where there have been increasing numbers of papers published in the scientific literature from multiple centres looking at possible associations between such exposure and changes in the epigenome [6]. Despite the volume of such work, there are still unanswered questions about child abuse and neglect and health and psychosocial outcomes. For example, in abused and neglected individuals, what are the associations between biological factors (e.g. epigenetic modifications of DNA) and psychological factors (e.g. suicidality)? What is the impact of the social world of the child on risk and resilience in the context of abuse and neglect? At which developmental periods are abuse and neglect most likely to increase risk of negative mental and physical health outcomes? What is the impact of different forms of abuse at different critical periods in development? Does this differ by gender?

Additionally, there continues to be issues with the quality of some work in the area. For example, much work is based on retrospective reports of ACE exposure from adults with a

relative paucity of high quality longitudinal prospective studies beginning in childhood [5, 6]. This is particularly concerning since there is poor agreement between retrospective and prospective reports of abuse and neglect [7].

Given the ~~apparent~~ complexity in relationships between potential causes, confounders, and outcomes of exposure to abuse and neglect, it ~~would be helpful~~ to consider the full spectrum of influences and exposures which a child may experience, in addition to abuse and neglect, as well as the interplay between biological and social factors, in order to develop a coherent model that is informative about mechanisms. This model may allow us to understand the relationship between the development of subsequent physical, psychological, and social manifestations of abuse and neglect and, perhaps more importantly, understand why some children seem to be protected from such negative outcomes. In this developing area of understanding, it is likely that interdependent processes will interact at multiple levels: from genetics, through the stress and immune systems, the brain, and into the family and wider community, with the potential for reciprocal influences and bidirectional causality at all levels [8].

Due to the increasing diversity of outcomes under examination, and the wide range of quality in the published literature, it has become important to find a way to better conceptualise and integrate this broad evidence base. This will enable researchers to better understand what evidence can be relied upon, and therefore what is known about the likely causes and outcomes of childhood abuse and neglect, how these might interact, and what this tells us about likely mechanisms. This is where the concept of “hallmarking” might be useful. The hallmarking technique was first applied to cancer studies, at a time when this literature was also experiencing a significant growth in volume and complexity [9]. The purpose of hallmarking is to find common factors by seeking commonalities across different studies and in most (if not all) types of cancer.

Applying this concept to the study of childhood abuse and neglect, we wanted to identify hallmarks across the entire biopsychosocial environment of the child and to consider the volume and quality of evidence for each of these. Recent theoretical models have focussed on the human stress response system as the "control centre" for human adaptation to severe stresses such as abuse and neglect. These suggest that only a truly integrated approach that involves all bio-ecological levels has the potential to identify mechanisms [10] . Some hallmarking processes have examined commonalities across both humans and other species [9, 11, 12] but we did not think that was appropriate here: whilst there are animal models of early life stress, we chose to look more specifically at abuse and neglect as opposed to early life stress more broadly. Animal models cannot distinguish these.

Many thousands of papers have been written about factors associated with child abuse and neglect and many literature reviews have been conducted exploring these. In order to bring together such a large body of literature, we have conducted a 'review of reviews' [13] as the first stage of our hallmarking process, followed by a synthesis of the findings of these with reference to the bio-ecological model. We aimed to answer the following questions:

1. What are the hallmarks of exposure to childhood abuse and neglect across the bio-ecological spectrum?
2. What is the strength of evidence to support each hallmark so identified?
3. What are the research gaps in this field, in terms of areas where further research or better quality research is needed?

Methods

The systematic review was performed in accordance with PRISMA, PRIMSA checklist is available in supplementary materials (S1). Studies were identified by searching the following electronic databases from 2009 to present: Ovid Medline ALL (R) (1946 to Present), OVID

Embase Classic & Embase (1947 to Present), OVID PsycInfo (1806 to Present) and the Cochrane Database of Systematic Reviews. All searches were run on 29th May 2019.

The search strategy was developed by a Subject Specialist Librarian in consultation with the review group. The final draft Medline search strategy was peer reviewed by another librarian not involved in the review. The search strategy utilised a combination of subject headings and keywords; the strategy was adapted to each database as required to take account of differences in subject headings and search tools. Due to time constraints a systematic review search filter was applied to the search strategy to maximise specificity. The search filters were developed by the Health Information Research Unit at McMaster University, Canada [14-16]. In addition, the results were limited to English Language and, because we wanted to focus on the recent literature, more likely to evidence current theoretical models, a publication date limit was set of within the last ten years (2009 to May 2019). The master search strategy for OVID Medline ALL (R) can be found in the supplementary material (S2).

The search strategy consisted of eight individual concepts drawn from the review question; these were searched individually and then combined to find relevant studies. The first search concept was 'child abuse & neglect' and the search terms included child abuse, childhood sexual abuse, child neglect and adverse childhood experiences. The second search concept was 'social factors', the search terms included socioeconomic factors, poverty, gender, sexuality, educational status and social support. The third search concept was 'genetic phenomena' and the search terms included genetics, epigenetics and biomarkers. The fourth concept was 'mental health', search terms included mental disorders, suicide, depression and PTSD. The fifth concept was 'physical health' and the search terms included obesity, smoking, heart disease and diabetes. The sixth search concept was 'stress responsivity'; the search terms included autonomic nervous system, stress response and heart rate. The seventh search concept was 'neuro-anatomical factors', the search terms include neuroimaging and

MRI. The final search concept was ‘inflammatory/endocrine markers’, the search terms included endocrine and immune biomarkers.

The PRISMA flow diagram [17] is shown in Figure 1. The total number of articles returned from the original search was 2255 and following removal of duplicates 1433 articles remained. 1433 records underwent title and abstract review for inclusion using inclusion / exclusion criteria agreed prior to the search process by at least 2 raters (see figure 2 for inclusion / exclusion criteria). Where conflict existed, this was resolved in a conference of the authors. Following this process, 269 articles were read in full by at least two reviewers. A further 90 records did not meet inclusion criteria when read in full and were excluded (reasons in Figure 1). All exclusions where checked and agreed by at least two authors. This left 178 articles which were data extracted and rated for quality using the AMSTAR checklist [18]. All articles were independently rated by at least two authors and discrepancies resolved at conference. Data on study setting, type of abuse, number of studies, and results were extracted.

Figure 1 PRISMA Flow Diagram

Figure 2 Inclusion and Exclusion Critera

We considered quantitative analysis using a network approach but this was not possible due to the wide range of variables examined in the reviews. Instead results are presented in narrative format.

Results

Characteristics of studies

One hundred and seventy nine studies were included. 43% of these studies were meta-analyses (n=77) and 57% were systematic reviews (n=102).

The review included studies from North America, Europe, South America, Asia, Africa and Australasia, see Figure 3.

Figure 3. Country of origin of studies included in reviews. (Details available on request)

Not all studies identified the sources of the studies (n=15), and many reviews and meta-analyses included studies from more than one country. Some studies used phases such as 'non-US high income countries' or 'industrialised countries', others grouped countries by continent, albeit not consistently.

Overall, we identified 4 broad categories: *Mind & Body*, *Genes & Epigenetic factors*, *Social Factors*, and *Biochemical Factors*. The distribution of papers by category is shown in Table 1.

Table 1. Types and numbers of papers sorted by thematic category and subthemes.

CATEGORY	SUBTHEME	BIO-ECOLOGICAL LEVELS	NUMBER OF PAPERS
• **BIOCHEMICAL FACTORS**		Microsystem	7
• **GENES AND EPIGENETIC FACTORS**		Microsystem	11
• **MIND AND BODY**			

	Individual mental Health, substance use and misuse	Microsystem	74
	Brain structure, neurodevelopment, cognition and personality	Microsystem	18
	Physical health	Microsystem	17
• **SOCIAL FACTORS**			
	Environmental risk factors	Exo- & Macrosystem	18
	Offending and antisocial behaviour	Meso- , Exo- & Macrosystem	10
	Relationships, parenting, sexual behaviour	Mesosystem	16
	Education/adults economic status	Meso- , Exo- & Macrosystem	2
	Resilience factors	All systems	6

‘*Biochemical factors’* included studies of markers of inflammation, the immune system, cortisol and other biomarkers. *‘Genes and Epigenetic factors’* looked at genetic and epigenetic markers. Due the number of papers in the categories *‘Body and Mind’* and *‘Social Factors’*, these were further divided into subthemes. *Body and Mind*: Mental Health and

Substance Use/Misuse'; 'Physical Health; 'Brain structure, neurodevelopment, cognition and personality'; *'Social Factors'*: 'Relationships, parenting, sexual behaviour' and 'Offending and antisocial behaviour'.

One hundred and forty-two studies (79%) investigated a combination of types of abuse and neglect, whereas thirty-three (18%) concentrated on sexual abuse, two (1%) on physical abuse and three (2%) on emotional abuse. The modal number of included studies per review was 12 (range 2-393).

Quality of the Studies

Overall, over two thirds (70%, n=126) of papers were rated as low or critically low quality. Just over a quarter (27%, n=46) were rated as moderate quality and only 3% (n=6) were rated as high quality (see figure 6). Most lower ratings could be explained by the lack of a 'risk of bias assessment' or a failure to incorporate such an assessment into the synthesis. Further, many papers lacked a rigorous search strategy and data extraction procedure.

To achieve a high-quality rating according to the AMSTAR rating scale, a paper needed to complete the search and data extraction in duplicate, i.e. by two independent authors, and have a rigorous 'risk of bias assessment' examining factors such as representativeness of the sample, loss to follow up etc. It also needed to investigate heterogeneity of outcome measures and publication bias.

No systematic reviews achieved high quality rating, however 6 meta-analyses did. Figure 3 shows the quality rating of articles by review type and thematic category.

Figure 3 Overview of AMSTAR ratings by type of review and theme

Findings of high-quality studies

Six papers received a high-quality AMSTAR rating. All were meta-analyses. 5 of these papers fell into the thematic category of Mind and Body and one into the category of Social Factors. Of the five papers in the Mind and Body category, four had the subtheme of 'mental health and substance use'. All of these papers investigated more than one type of abuse. Details of these papers are summarised in Table 2.

Table 2.

Author	Year	Category	AMSTAR score	Type of abuse investigated	Number of included studies	Number of participants in meta-analysis	Countries of studies	Summary of result
Bailey T	2018	Body & Mind	High	Sexual abuse, physical abuse, emotional abuse, physical neglect, emotional neglect	41(29)	4680	Not stated	• Sexual abuse and neglect affect severity of hallucination<br>• Sexual abuse, physical neglect, and emotional neglect are associated with delusion severity<br>• Sexual abuse affected severity of positive symptoms<br>• Emotional neglect and physical neglect are associate

								with severity of negative symptoms
Castellvi, P.	2017	Body & Mind	High	Child maltreatment	26	143,730	Netherland, New Zealand, United States, Norway, Canada, United Kingdom, Denmark, Finland	• Physical abuse increases risk of suicidal behaviour
Fusar-Poli, P	2017	Body & Mind	High	Childhood abuse,	44	Not stated	Not stated	• Ultra-high risk state for psychosis is associated with

				childhood neglect				physical neglect, and emotional abuse
Jones, L	2012	Body & Mind	High	physical violence, sexual violence, emotional abuse, neglect and any combination of those	17	18,374	United States, United kingdom, Sweden, Finland, Spain, Israel	• Children with disabilities are at higher risk of physical, emotional and sexual abuse, and neglect.
Normal, R.E	2012	Body & Mind	High	Physical abuse, Emotional	124	Not stated	Australia, New Zealand, Western	• Physical abuse, and emotional abuse increases risk for depressive disorder, anxiety disorders and eating disorders

	abuse, neglect	Europe, North America	• Physical abuse, and neglect doubled odds of childhood behaviour and conduct disorder<br>• Physical abuse, and neglect increased risk of alcohol misuse and dependence<br>• Physical abuse, emotional abuse, and neglect increased risk of suicidal behaviour<br>• Physical abuse, emotional abuse, and neglect were associated with increased risk of STI (including HIV) and increased risky sexual behaviour

								• Physical abuse, emotional abuse, and neglect increased the risk of smoking and being obese
Winokur M.	2014	Social Factors	High	Abuse, Neglect	102	666,615	United States, Spain, Norway, Ireland, Israel, Sweden, the Netherlands, Australia	• Kinship care mediates the relationships between childhood abuse and mental health

Fusar-Poli et al. [19] performed a systematic review and meta-analysis of environmental factors associated with ultra-high risk for psychosis, including childhood abuse and neglect. Forty-four studies were included in their review. This review only included papers written in English and did not report the origin of original studies. They found strong evidence that emotional abuse (OR=5.843, 95% CI 1.794-19.027) and physical neglect (OR=3.066, 95% CI 1.043-9.013) experienced during childhood are associated with ultra-high-risk state for psychosis;

Bailey et al. [20] studied the association between childhood trauma and severity of hallucinations and delusions in psychotic disorders. Their review included 41 studies, of which 29 were included in the meta-analysis with 4680 participants in total. This review defined childhood trauma to include sexual abuse, physical abuse, emotional abuse, physical neglect, emotional neglect, and bullying. The countries of origin of the included studies were not stated, however only studies published in English were included. They found that childhood sexual abuse and neglect was significantly correlated with severity of hallucinations ($r=.172$, $p<0.001$). Sexual abuse and physical or emotional neglect was also associated with delusion severity ($r=.199$, $p<0.001$). Further, sexual abuse increased severity of positive symptoms and negative symptoms of schizophrenia were associated with childhood neglect.

Castellvi et al. [21] investigated the association between exposure to violence and risk for suicide. The meta-analysis included 26 papers with a total sample of 143,730. Violence was defined as child maltreatment, bullying, dating violence and community violence. The included studies originated in the Netherland, New Zealand, United States, Norway, Canada, United Kingdom, Denmark, Finland. They found participants with experience of physical abuse to have an increased risk of suicidal behaviour (OR=2.25; 95% CI: 1.85-2.73). The evidence was weaker for the association between sexual abuse and suicide behaviour. There

were not enough studies that investigated the link between emotional abuse and suicide behaviour. The association between neglect and suicide behaviour was not significant.

Norman et al. [22] investigated a range of associations with health outcomes and physical abuse, emotional abuse and neglect. These consequences were not limited to mental health, and included HIV risk and obesity. However, most included papers were about mental health and substance use. The studies included in this review originated in Australia, New Zealand, Western Europe and North America. They found that adults who were physically abused (OR=1.54, 95% CI 1.16-2.04), emotionally abused (OR=3.06, 95%2.43-3.85) or neglected (OR=2.11, 95% CI 1.61-2.77) were at higher risk of developing depressive disorders, anxiety disorders and eating disorders. The association between depression and physical abuse was only significant in high-income countries and not in low- and middle-income countries. However, the association between neglect and depression was the same across countries. Physical abuse and neglect were also associated with double the odds of developing behavioural and conduct disorders during childhood. Suicidal behaviour increased with exposure to physical and emotional abuse, as well as neglect. They also found a higher risk of alcohol misuse and dependence and to a lesser extent drug use. They found an increase in risky sexual behaviours and Sexually Transmitted Infections (physical abuse OR=1.78, 95% CI 1.50-2.10; emotional abuse OR=1.75, 95% CI- 1.49-2.04; neglect OR=1.57, 95% CI 1.39-1.78).There was an increased risk of current smoking associated with a history of emotional (OR=1.70, 95% CI 1.55-1.87) and physical (OR=1.55, 95% CI=1.09-2.21) abuse; and an increased risk of obesity associated with physical (OR=1.32, 95% CI 1.06-1.64) and emotional (1.24, 95% CI 1.13-1.36) abuse. The evidence for other associations with physical health problems, such as cardiovascular disease and cancer, was weak.

Jones et al. [23] reviewed the risk of violence against children living with disabilities. Of the 17 papers that are included in the meta-analysis, 11 included risk estimates and 16 included

prevalence rates of violence exposure. The sample sizes were 13,505 children and 14,721 children, respectively. Violence was defined as physical violence, sexual violence, emotional abuse, neglect and any combination of those. They founded that children with disabilities were at increased risk of abuse and neglect in comparison to non-disabled children (OR=3·68, 95% CI 2·56–5·29). The pooled prevalence of violence against children with disabilities was 26.7% (95% CI 13.8-42.1); this analysis did not include a control group to allow comparison of the prevalence of violence exposure in disabled versus non-disabled children. There were high levels of heterogeneity due to type of reporting, study setting and type of disability.

Winokur et al. [24] was the only high quality paper not in the 'Body & Mind' category. The authors reviewed papers that compared outcomes for children removed from home due to abuse or neglect who were subsequently placed in kinship care versus non-kin foster care. 102 papers were included, with a total number of 666,615 children. Most of the included studies were conducted in the USA, with the rest conducted in Spain, Norway, Ireland, Israel, Sweden, the Netherlands and Australia. They reported that children placed in kinship care after suffering abuse or neglect had fewer behavioural problems (standardised mean difference= -0.33, 95% CI -0.49 to -0.17), fewer mental health disorders (OR=0.51, 95% CI 0.42-0.62) and better wellbeing (OR=0.50, 95% CI 0.38- 0.64), than children placed in non-kin foster care.

The findings of these high-quality papers are mapped onto the bio-ecological model in Figure 4.

Figure 4. Model of interactions of factors. Key- 1) Bailey et al 2) Castellvi et al 3) Fusar-Poli 4) Jones et al 5) Norman et al 6) Winokur et al

Findings of medium quality studies

There were 46 medium quality papers. Within the ‘Mind and Body’ category 20 had the subtheme ‘Mental Health and Substance Use and Misuse’, eight had the subtheme ‘Physical Health, and one had the subtheme ‘Brain Structure, Neurodevelopment, Cognition and Personality’. In the category ‘Social Factors’, six papers explored the subtheme ‘Relationships, Parenting, Sexual Behaviour’ and four studied the theme ‘Offending and Antisocial Behaviour’. ‘Environmental Risk Factors’ were investigated by two studies. The subthemes ‘Resilience Factors’ and ‘Education/ Adult Economic Status’ had one study each. Thirty-two papers investigated more than one type of abuse or neglect, 12 concentrated on sexual abuse, one on physical abuse and one on emotional abuse. The quality rating of these studies was mainly influenced by the lack of a rigorous risk of bias assessment, or a failure to include the assessment outcome in the analysis of results.

A supplementary table (S3) includes a summary of the papers, including suggestive findings.

Discussion

From our review of the literature, we can confidently identify five hallmarks of abuse and neglect in childhood.

1. Increased risk of psychopathology;

2. Increased risk of obesity;

3. Increased risk of participating in high risk sexual behaviours

4. Increased risk of smoking.

5. Increased risk of abuse and neglect in children with disabilities

There is ample evidence for poorer physical and mental health outcomes for adults who have experienced abuse and neglect and we tentatively suggest that smoking, obesity and possibly risky sexual behaviours might be mediators for this. Certainly smoking and obesity are associated with a number of physical impairments and premature death [25].

There may also be an argument that resilience could be a sixth hallmark. In all the studies included in this review, there were participants who had been exposed to the study condition who had not developed the measured behaviours or outcomes. However given that there was no direct high quality study looking at resilience per se it is not possible to say if these children had in fact experienced no long term negative outcome from their exposure to abuse and neglect, or if certain adverse outcomes which may have been present had simply not been measured. There is a need for researchers to consider designing high quality studies which examine resilience directly as a carefully defined and measured outcome variable.

There is good evidence that having a disability is a risk factor for experiencing abuse and neglect [23] This is an important focus for future research: it is often assumed that developmental problems are the *result* of abuse and neglect, but we have found this not to be the case, at least for symptoms of neurodevelopmental problems such as ADHD and Autism [26, 27].

There may be a mediating effect on the outcomes of abuse and neglect in children who are placed in kinship care versus foster care, although there is insufficient evidence to state this categorically since children placed in kinship versus non-relative foster care could have had different levels of psychopathology in the first place. Further research in this area is suggested.

Despite the burgeoning number of studies on the effects of abuse and neglect in childhood, there remains a fundamental issue with the quality of much of the literature, across both

systematic reviews and meta-analysis. Of the 178 studies included in this review only 3% were rated as high quality using the AMSTAR tool and only a further 27% managed a moderate quality rating.

There is currently no agreed standard with relation to how studies report their exposures and outcomes. For example, in considering the types of abuse and neglect (or adverse childhood experiences more widely) which study participants have been exposed to, some authors report this precisely, allowing for replication in further studies, however many do not. This makes synthesis of outcome findings challenging if not impossible and decreases the likelihood of findings emerging consistent with types of abuse and neglect. Adoption of an agreed standard in terms of the reporting of exposure to abuse and neglect in study participants, and of commonly-measured outcomes, would help increase the quality of future meta-analysis, and perhaps make possible a network study which may help unravel the complexity of the underlying interactions between variables.

Similarly, there remains a common difficulty in establishing causal relationships between abuse and neglect and outcomes, associated with the frequent reliance on retrospective adult self-reports of childhood abuse and neglect. Baldwin and colleagues [7] have shown that agreement between prospective and retrospective measures of abuse is poor.

A major oversight in the extant research on abuse and neglect is the fact that we were able to find no review linking different factors across domains or considering multiple levels of the bio-ecological model. The potential for interactions between factors across domains is therefore not addressed at all in the large number of "silo" studies reviewed here. A high level of complexity is inevitable when biological systems relevant to abuse and neglect have such diverse purposes, components and actions, yet are intimately related in their functioning - as is true, for example, for the hypothalamic-pituitary-adrenal-axis and the immune system.

Methodologies adapted for complex systems are therefore crucial if we are to advance in this field. For more information on this see Ioannidis and colleagues [11]. Questions such as "how do physical factors affect mental health factors?" are not considered. Of the papers reviewed, only one high quality review looked at mediating or moderating factors (kinship care) that might link childhood abuse and neglect with outcomes.

We found no high-quality reviews considering the potential impact which social relationships (either positive or negative) might have on the manifestations of effects of abuse and neglect. This may be a challenging area in which to work as social relationships could be seen as cause, confounder and outcome. The same would be true of other outer aspects of the Bio-Ecological model such as the impact of social policy and state actions. We can make no comment on the effects of the outer layers of the bio-ecological model since there is virtually no evidence available, here, at present. This area has not been studied in any detail and requires further consideration from researchers.

Examining the gaps, there is clearly a need for future researchers in this field to consider study designs that embrace complexity if crucial unanswered questions, especially about causality and mechanisms, are to be addressed. This is no truer than around the question of resilience. Given the lack of focus on resilience the reviews we have examined, we are not able to answer any questions in relation to how to prevent adverse outcomes in children exposed to abuse and neglect. This is an area of research which we would argue requires urgent attention.

Finally, there were no studies which reliably addressed the potential significance of the timeline of exposure to abuse and neglect in relation to the developmental stage of the child. For example, questions have not been answered regarding whether there are ages or stages of

development during which there is more or less risk for the development of certain outcomes of abuse and neglect.

This study aimed to elucidate hallmarks of abuse and neglect robustly evidenced across multiple high-quality studies. In terms of limitations, our hallmarks are confined to human studies rather than across taxa as in the hallmarking work on aging and cancer. Whilst there are animal models of early life stress, we were looking more specifically at effects of abuse and neglect which is not readily distinguished from other sources of early stress in animal models. Secondly, our conclusions are based on the quality of systematic review articles and meta-analyses rather than on the underlying primary research. There might be undetected hallmarks based on high quality individual studies that we missed because they haven't been systematically reviewed or because the systematic review was poorly done. Our search was limited to articles in English, and by limiting our search to systematic reviews we may have omitted relevant findings in the "grey literature". We did consider undertaking a network analysis however this was not possible due to the heterogeneity of outcomes and study parameters. Indeed, this heterogeneity may also have impacted our identification of hallmarks since it is likely to have limited the potential for meta-analysis.

Conclusions

We believe that we have, for the first time, demonstrated five hallmarks of abuse and neglect. It may be that resilience represents a sixth hallmark however further research is required to confirm this.

There are clear gaps in the literature, for example there is little research on certain biological factors and virtually no research on wider societal factors such as the quality of neighbourhoods. These gaps must be addressed if progress is to be made in understanding

the impact, and mechanisms of impact, of abuse and neglect and, perhaps more importantly, understanding how to protect abused and neglected children from adverse outcomes.

Using study designs that embrace complexity, in order to examine inter-relationships within and across the bio-ecological model, is likely to be key in answering some of these outstanding questions. Future studies need to be adequately designed and powered to achieve this.

Acknowledgements

We are grateful for the contributions of Evi Bali, Encrico Venturini, Emily Roberts, Pablo Barrera, Lilliane Bills, Makhib Choudkhuri, Orla Macpherson, and Rachel Whyte to the double rating of papers. We are also grateful to Irene O’Neill for administrative support.

References

1. Felitti VJ, Anda RF, Nordenberg D, Williamson DF, Spitz AM, Edwards V, et al. Relationship of Childhood Abuse and Household Dysfunction to Many of the Leading Causes of Death in Adults: The Adverse Childhood Experiences (ACE) Study. American Journal of Preventive Medicine. 1998;14(4):245-58. doi: 10.1016/S0749-3797(98)00017-8.

2. Ashton K, Bellis M, Hughes K. Adverse childhood experiences and their association with health-harming behaviours and mental wellbeing in the Welsh adult population: a national cross-sectional survey. The Lancet. 2016;388:S21-S. doi: 10.1016/S0140-6736(16)32257-7.

3. Mobilizing Action for Resilient C. Mobilizing Action for Resilient Communities through policy and advocacy: A toolkit for trauma-informed, cross-sector networks. Philadelphia, PA: Health Federation of Philadelphia, 2019 Contract No.: Report.

4. Nemeroff Charles b. Paradise Lost: The Neurobiological and Clinical Consequences of Child Abuse and Neglect. Neuron. 2016;89(5):892-909. doi: 10.1016/j.neuron.2016.01.019.

5. Lacey RE, Minnis H. Practitioner Review: Twenty years of research with adverse childhood experience scores – Advantages, disadvantages and applications to practice. Journal of Child Psychology and Psychiatry. 2020;61(2):116-30. doi: 10.1111/jcpp.13135.

6. Lang J, McKie J, Smith H, McLaughlin A, Gillberg C, Shiels PG, et al. Adverse childhood experiences, epigenetics and telomere length variation in childhood and beyond: a systematic review of the literature. European child & adolescent psychiatry. 2019. doi: 10.1007/s00787-019-01329-1.

7. Baldwin JR, Reuben A, Newbury JB, Danese A. Agreement Between Prospective and Retrospective Measures of Childhood Maltreatment: A Systematic Review and Meta-analysis. JAMA psychiatry. 2019;76(6):584. doi: 10.1001/jamapsychiatry.2019.0097.

8. Bronfenbrenner U. Making human beings human: Bioecological perspectives on human development. Bronfenbrenner U, editor. Thousand Oaks, CA: Sage Publications Ltd; 2005. xxix, 306-xxix, p.

9. Hanahan D, Weinberg Robert a. Hallmarks of Cancer: The Next Generation. Cell. 2011;144(5):646-74. doi: 10.1016/j.cell.2011.02.013.

10. Del Giudice M, Ellis BJ, Shirtcliff EA. The Adaptive Calibration Model of stress responsivity. Neuroscience and biobehavioral reviews. 2011;35(7):1562-92. doi: 10.1016/j.neubiorev.2010.11.007.

11. Ioannidis K, Askelund AD, Kievit RA, Van Harmelen A-L. The Complex Neurobiology of Resilient Functioning After Child Maltreatment. BMC Medicine. 2020;(In Press). doi: 10.1186/s12916-020-1490-7.

12. Kalisch R, Cramer AOJ, Binder H, Fritz J, Leertouwer I, Lunansky G, et al. Deconstructing and Reconstructing Resilience: A Dynamic Network Approach. Perspectives on Psychological Science. 2019;14(5):765-77. doi: 10.1177/1745691619855637.

13. Gough D, Oliver S, Thomas J. An introduction to systematic reviews. Second edition.. ed: London : SAGE; 2017.

14. Montori VM, Wilczynski NL, Morgan D, Haynes RB. Optimal search strategies for retrieving systematic reviews from Medline: analytical survey. British Medical Journal Publishing Group; 2005. p. 68.

15. Wilczynski NL, Haynes RB. EMBASE search strategies achieved high sensitivity and specificity for retrieving methodologically sound systematic reviews. Journal of clinical epidemiology. 2007;60(1):29-33. doi: 10.1016/j.jclinepi.2006.04.001.

16. Eady AM, Wilczynski NL, Haynes RB. PsycINFO search strategies identified methodologically sound therapy studies and review articles for use by clinicians and researchers. Journal of clinical epidemiology. 2008;61(1):34-40. doi: 10.1016/j.jclinepi.2006.09.016.

17. Moher D, Liberati A, Tetzlaff J, Altman DG. Preferred reporting items for systematic reviews and meta-analyses: the PRISMA statement. British Medical Journal Publishing Group; 2009.

18. Shea BJ, Reeves BC, Wells G, Thuku M, Hamel C, Moran J, et al. AMSTAR 2: A critical appraisal tool for systematic reviews that include randomised or non-randomised studies of healthcare interventions, or both. BMJ. 2017;358:j4008. doi: doi:10.1136/bmj.j4008

19. FusarPoli P, Tantardini M, De Simone S, RamellaCravaro V, Oliver D, Kingdon J, et al. Deconstructing vulnerability for psychosis: Meta-analysis of environmental risk factors for psychosis in subjects at ultra high-risk. Psychiatrie & Psychobiologie. 2017;40:65-75. doi: http://dx.doi.org/10.1016/j.eurpsy.2016.09.003.

20. Bailey T, AlvarezJimenez M, GarciaSanchez AM, Hulbert C, Barlow E, Bendall S. Childhood trauma is associated with severity of hallucinations and delusions in psychotic disorders: A systematic review and meta-analysis. Schizophrenia bulletin. 2018;44(5):1111-22. doi: http://dx.doi.org/10.1093/schbul/sbx161.

21. Castellvi P, Miranda-Mendizabal A, Pares-Badell O, Almenara J, Alonso I, Blasco MJ, et al. Exposure to violence, a risk for suicide in youths and young adults. A meta-analysis of longitudinal studies. Acta Psychiatrica Scandinavica. 2017;135(3):195-211. doi: https://dx.doi.org/10.1111/acps.12679.

22. Norman RE, Byambaa M, De R, Butchart A, Scott J, Vos T. The long-term health consequences of child physical abuse, emotional abuse, and neglect: a systematic review and

meta-analysis. PLoS Medicine / Public Library of Science. 2012;9(11):e1001349. doi: https://dx.doi.org/10.1371/journal.pmed.1001349.

23. Jones L, Bellis MA, Wood S, Hughes K, McCoy E, Eckley L, et al. Prevalence and risk of violence against children with disabilities: a systematic review and meta-analysis of observational studies. Lancet. 2012;380(9845):899-907. doi: https://dx.doi.org/10.1016/S0140-6736(12)60692-8.

24. Winokur M, Holtan A, Batchelder KE. Kinship care for the safety, permanency, and well-being of children removed from the home for maltreatment. Cochrane Database of Systematic Reviews. 2014:(1)-2014 Jan 31. doi: https://dx.doi.org/10.1002/14651858.CD006546.pub3.

25. Freedman DM SA, Rajaraman P, Doody MM, Linet MS, Ron E. The Mortality Risk of Smoking and Obesity Combined. American Journal of Preventive Medicine. 2006;31(5):355-62.

26. van Der Kolk B, Ford JD, Spinazzola J. Comorbidity of developmental trauma disorder (DTD) and post-traumatic stress disorder: findings from the DTD field trial. European Journal of Psychotraumatology. 2019;10(1). doi: 10.1080/20008198.2018.1562841.

27. Dinkler L, Lundström S, Gajwani R, Lichtenstein P, Gillberg C, Minnis H. Maltreatment- associated neurodevelopmental disorders: a co- twin control analysis. Journal of Child Psychology and Psychiatry. 2017;58(6):691-701. doi: 10.1111/jcpp.12682.

Supplementary Materials

S1: Supplementary Data- PRISMA Checklist

S2: Supplementary Data- Detailed Search Strategy

S3: Supplementary Table- Details of medium quality articles

Figure 1- PRISMA Flow Diagram

Click here to download Figure Figure 1!.tif

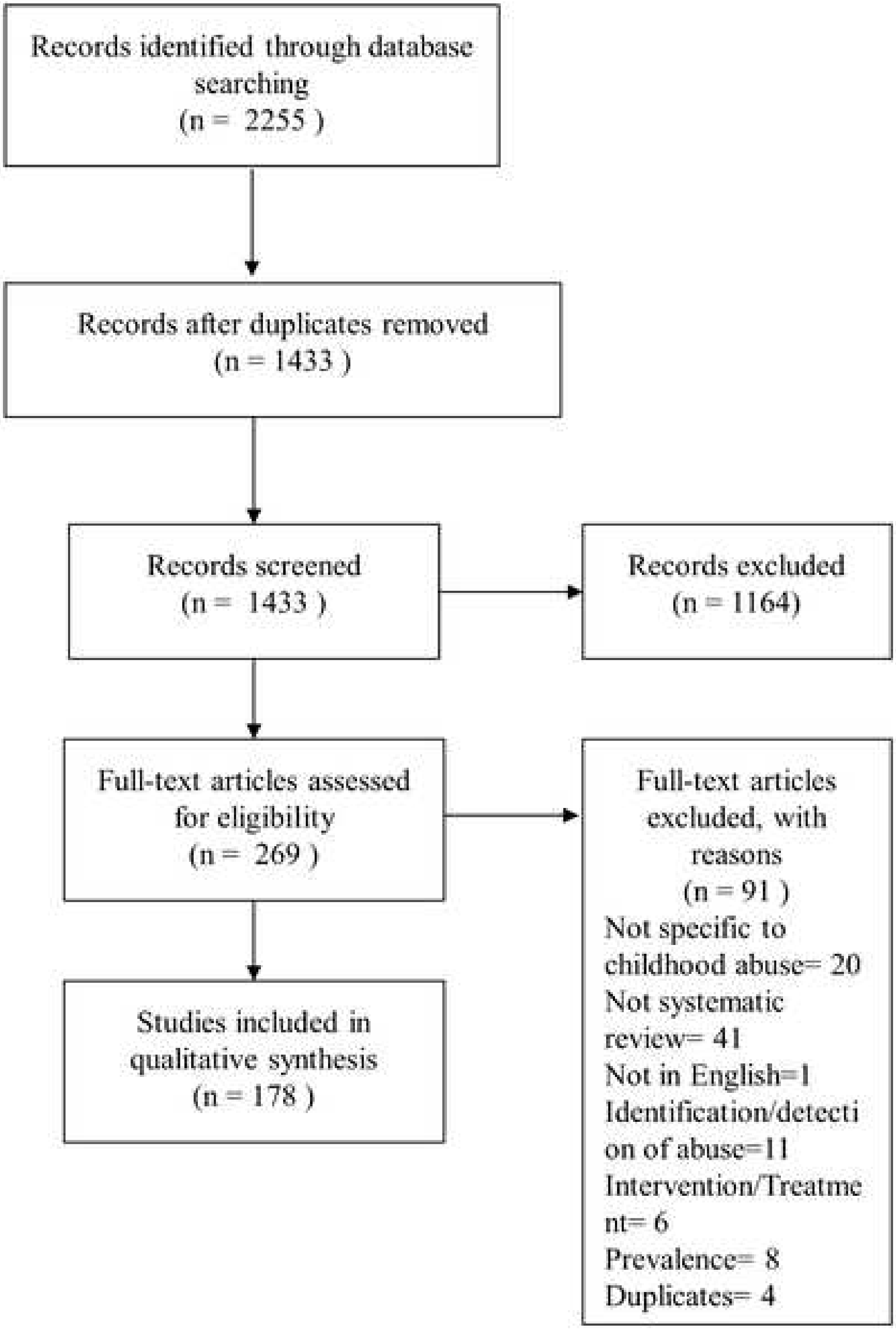

Figure 2

Click here to download Figure Figure 2.tif

Inclusion Criteria	Exclusion Criteria
Systematic Review or Meta-analysis	Original Articles, Conference Articles, Abstracts, Posters
Documented abuse or neglect (Physical abuse, emotional abuse, sexual abuse, neglect, or a combination)	No documented abuse.
Measures a potential cause or consequence of abuse or neglect	Studies purely concerned with the prevalence, detection, prevention or treatment of abuse which do not consider potential causes or consequences.
Human Participants	Animal Studies
Quantitative Studies (can be mixed qualitative / qualitative)	Qualitative Studies
Published since 2009	Published before 2009
English Language	Not English Language

Figure 3

Click here to download Figure Figure 3.tif

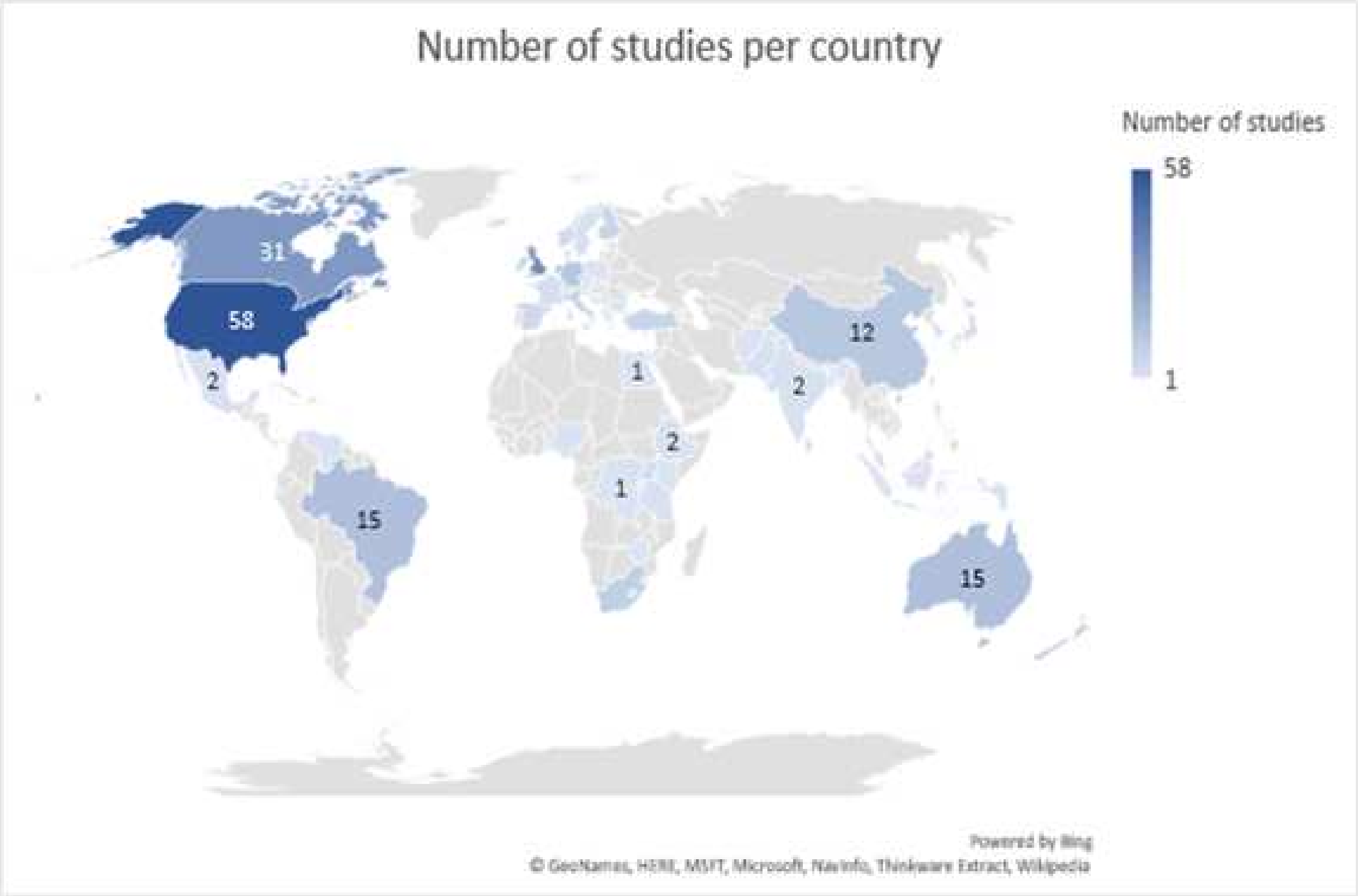

 Click here to download Figure Figure 4!.tif

Meta-analysis total	N=76	Systematic Review	N=102
High Quality	n=6 (7.9%)	High Quality	n=0 (0%)
Medium Quality	n=22 (28.9%)	Medium Quality	n=24 (23.5%)
Low Quality	n=48 (63.1%)	Low Quality	n=78 (76.5%)

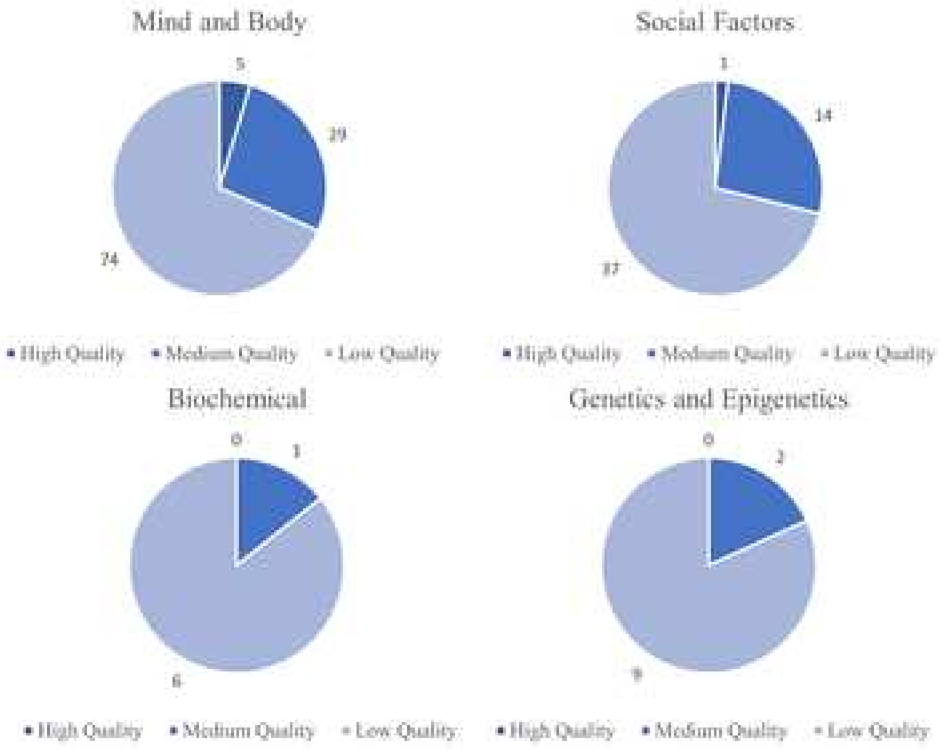

Figure 5 Click here to download Figure Figure 5.tif

Modelling of identified risk factors and the bio-ecological model.

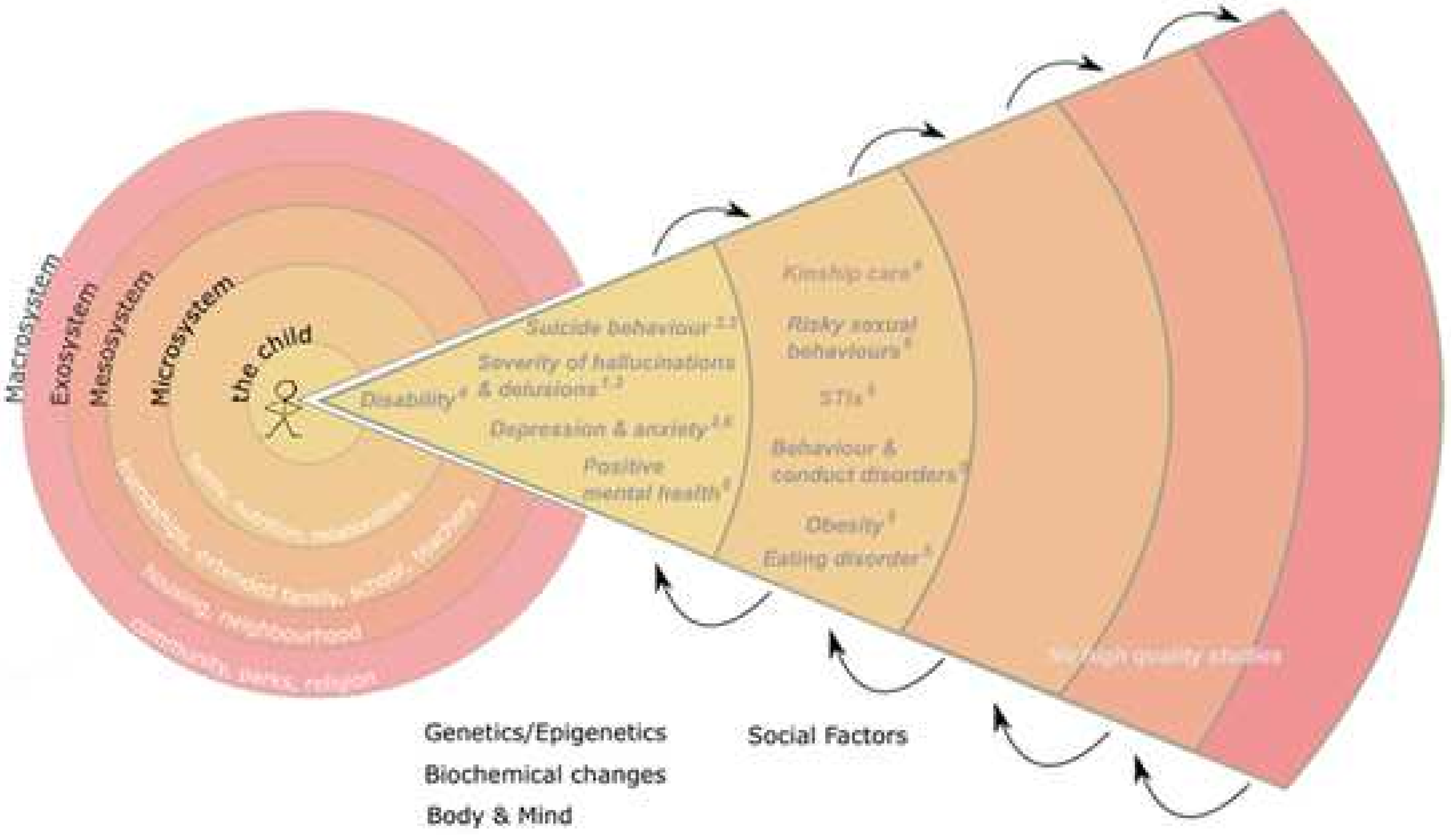

S1- PRISMA Checklist

Click here to access/download
Supporting Information
S1.doc

S2- Detailed Search Strategy

Click here to access/download
Supporting Information
S2.docx

S3- Supplementary Table

Click here to access/download
Supporting Information
S3.docx
