# Supplementary material for: The hallmarks of childhood abuse and neglect: A systematic review
Source: PLoS One. 2020 Dec 8;15(12):e0243639. doi: 10.1371/journal.pone.0243639 (PMC7723263; doi:10.1371/journal.pone.0243639)
Supplement: Attachment — Submitted filename: Response to Reviewers.docx [file pone.0243639.s006.docx]

Dr Daniel Kerr
Department of Academic Child Psychiatry
University of Glasgow
1 Horselethill Road
Glasgow G12 9LX
[Daniel.Kerr@glasgow.ac.uk](mailto:Daniel.Kerr@glasgow.ac.uk)

08/08/2020

RE: The hallmarks of childhood abuse and neglect: a systematic review
Ref: PONE-D-20-08783

Dear Colleagues,

Thank you for your considered comments on our manuscript “The hallmarks of childhood abuse and neglect: a systematic review”. Please find enclosed a revised version of our manuscript, which we have adapted in response to your comments. I would also like to directly address your comments below:

Reviewer 1:

We agree that our negative findings are likely to be of greater significance than the positive findings and have made changes to the content and emphasis of our discussion to address this. We also agree that questions raised in the introduction are not fully addressed by the review. Parts of the introduction have been re-written in response to other comments and as such some of these questions are no longer present; and where they are, we have been more explicit in addressing the inability of our review to answer them in our discussion.

In reference to further comments made:

1) The manuscript has been changed to use more consistent terminology around child maltreatment and to be more explicit around whether hallmarks are consequences or risk factors for CM. For clarity we are only addressing hallmarks of exposure to CM (be they risk factors for being exposed to CM, or consequences of exposure to CM). Perpetration of CM is only of relevance to our review if it is being studied as a consequence of CM (eg. are individuals exposed to CM at greater risk of perpetrating CM themselves?)
2) The introduction has been changed to be more explicit in our focus on CM and to explicitly distinguish CM from the wider concept of ACEs.
3) The introduction has been re-written in response to point 2 and as such some of these original points are no longer included. Where these questions are included we have been more explicit in the discussion about how our review was not able to answer these.
4) We have added a more detailed description of AMSTAR-2 in the methods section and have included the items included in AMSTAR-2 as a supplementary material.

Reviewer 2:

1) We are grateful for your comments on the draft and for highlighting some unfortunate typographical errors. Parts of the manuscript have been re-written in response to other comments; where the highlighted errors persist, we have corrected these.
2) We have included more details on the sub-themes in our results sections and referenced to a blank version of the bio-ecological model as suggested.
3) We have included a more detailed description of AMSTAR-2 in the methods section and added the items included in AMSTAR-2 as a supplementary material. In reference to the high-quality studies, AMSTAR-2 assigns a score of high-quality to studies with no, or one non-critical weakness. Not describing the countries of origins of included papers would be considered a non-critical weakness and such papers could be rated as high quality provided that no other weaknesses are present. The paper by Fusar-Poli included a range of sample sizes for individual analyses but did not give an overall sample size. The table has been amended to make this clearer.
4) The figure has been adapted to aid clarity.
5) The figures have been re-numbered. Thank you for pointing out this error.
6) We agree that all our findings must be interpreted in light of the specific methodology that has been employed. We have amended the specific phrase highlighted, and overall emphasis of the discussion in an effort to make this clearer.
7) An internal protocol was developed but was not published or registered. The supplementary materials have been amended to make this clear.

In addition to the issues highlighted by the reviewers we have also taken the opportunity of revising this article to make the following changes:
a) Inclusion of additional sections in the results and discussion to comment on the preponderance of studies occurring in WEIRD (Western, Educated, Industrialised, Rich and Democratic) countries and how this may impact the interpretation of research findings.
b) Changing the order in which high-quality articles are discussed in the results section to follow the order in which they are presented in table 2, in order to aid readability of the article.
c) In this cycle of the review process the editorial team have highlighted issues with the reproduction rights for figure 3 (the heat map of distribution of papers by country). We have been unable to secure creative commons agreement from the rights-holder and so have had to remove figure 3. We have renumbered the figures accordingly.

I am grateful for your contribution to the review process and I hope that these amendments are to your satisfaction.

Yours Sincerely,

Dr Daniel Kerr

Clinical Lecturer in Psychiatry
University of Glasgow
